# Snappy: fast identification of DNA methylation motifs based on oxford nanopore reads

**DOI:** 10.1093/bioadv/vbaf296

**Published:** 2025-11-21

**Authors:** Dmitry N Konanov, Danil V Krivonos, Vladislav V Babenko, Elena N Ilina

**Affiliations:** Laboratory of Mathematical Biology and Bioinformatics, Research Institute for System Biology and Medicine of Rospotrebnadzor, Moscow, 117246, Russia; Laboratory of Mathematical Biology and Bioinformatics, Research Institute for System Biology and Medicine of Rospotrebnadzor, Moscow, 117246, Russia; Moscow Center for Advanced Studies, Moscow, 123592, Russia; Department of Biomedicine and Genomics, Lopukhin Federal Research and Clinical Center of Physical-Chemical Medicine of Federal Medical Biological Agency, Moscow, 119435, Russia; Laboratory of Mathematical Biology and Bioinformatics, Research Institute for System Biology and Medicine of Rospotrebnadzor, Moscow, 117246, Russia

## Abstract

**Motivation:**

Nowadays, DNA methylation in bacteria is studied mainly using single-molecule sequencing technologies like PacBio and Oxford Nanopore. In nanopore sequencing, calling of methylated positions is provided by special models implemented directly in basecallers. Prokaryotic DNA methyltransferases are site-specific enzymes, which catalyze methylation in specific methylation motifs. Inference of these motifs is usually performed using third party software like MEME providing classical motif enrichment based only on sequence data. However, currently used motif enrichment algorithms rely only on sequence data, and do not use additional base modification information provided by the basecaller.

**Results:**

Herein, we present a new tool Snappy, which is actually rethinking of the original Snapper algorithm but does not use any enrichment heuristics and does not require control sample sequencing. Snappy combines basecalling data processing with a new graph-based enrichment algorithm, thus significantly enhancing the enrichment sensitivity and accuracy. The versatility of the method was shown on both our and external data, representing different bacterial species with complex and simple methylome.

**Availability and implementation:**

Source code and documentation is hosted on GitHub (https://github.com/DNKonanov/ont-snappy) and Zenodo (zenodo.org/records/16731817). For accessibility, Snappy is installable from PyPi using “pip install ont-snappy” command.

## 1 Introduction

In prokaryotes, DNA methylation is catalyzed by DNA methyltransferases (MTases), special site-specific enzymes (or enzymatic complexes) that modify nucleotide bases in dsDNA in specific nucleotide contexts (Gao *et al.* 2023). The most known modification types are methylation of adenine in 6th position, and methylation of cytosine in fourth or fifth positions, producing 6NmA, 5mC and 4NmC, respectively. Identification of genome contexts containing modified bases is primarily provided by sequencing technologies designed to analyze native DNA material, such as PacBio SMRT and Oxford Nanopore (Liu *et al.* 2022). Specifically, the most recent Oxford Nanopore r10 chemistry and new software significantly improved the quality of methylated bases identification (Galeone *et al.* 2025), thus allowing the analysis of DNA methylomes *de novo*, without sequencing control samples.

For the organisms for which the repertoire of active DNA MTases and corresponding methylation motifs is preliminary known, analysis of DNA methylation is quite trivial, and includes direct consideration of genome positions containing target motifs, and fraction of reads that bring a methylated base in these positions. On the other hand, while the specificity of methyltransferases is not known, it is first required to identify sequences of methylation motifs, which is usually done using motif enrichment algorithms such as MEME (Bailey *et al.* 2015) or STREME (Bailey 2021). However, the algorithms currently used for methylation motif identification rely only on genome contexts surrounding modified bases (in FASTA format), and do not use base modification information provided by the basecaller as auxiliary MM/ML tags in the BAM output.

Herein, we present Snappy, the first tool that combines motif enrichment with simultaneous analysis of basecalling results. As its predecessor Snapper (Konanov *et al.* 2023), Snappy is primarily oriented on Oxford Nanopore data, but unlike Snapper, it does not use any heuristics, does not require control sample sequencing, and is significantly easier to run. In this study, we validate Snappy on *Helicobacter pylori—*an organism with one of the most complex methylomes (Ailloud *et al.* 2023), and few other bacteria, and compare it with the MicrobeMod pipeline (Crits-Christoph et al. 2023). MicrobeMod uses STREME motif enrichment algorithm, but realizes an additional motif correction procedure, including conversion of any B, D, H, and V letters to N, or conversion any single base (A, T, G, C) to N if it does not cover more than 80% of modified contexts (https://github.com/cultivarium/MicrobeMod). Snappy uses a new graph-based enrichment algorithm and also conducts a motif correction procedure, but makes it based on modification probabilities assigned by Dorado to genome positions.

## 2 Implementation

### 2.1 Tool description

Snappy is a new tool designed for fast and accurate identification of DNA methylation motifs mainly based on Oxford Nanopore sequencing data. The algorithm uses an iterative approach, where each iteration consists of two steps: the first step is formation of a short anchor motif variant (up to six bases), and the second step is extending and correction of the anchor variant based on the modification probabilities assigned by basecaller for all genomic contexts satisfying the anchor motif or its close derivatives. The detailed description of the algorithm is available in Supplementary Data 1, available as supplementary data at *Bioinformatics Advances* online, some comments about Snappy behavior are listed in Supplementary Data 2, available as supplementary data at *Bioinformatics Advances* online. Since Snappy is a fully-automated tool, it does not require any user control during running, unlike specialized pipelines such as Nanodisco (Tourancheau *et al.* 2021).

**Figure 1.**
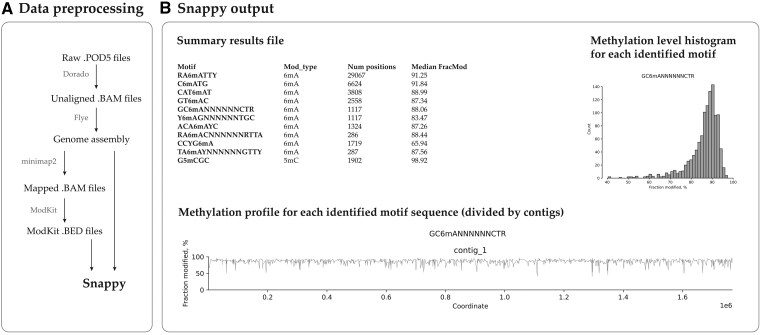
(A) Data preprocessing procedures required to prepare input files required for Snappy. (B) The main output provided by Snappy includes both text and graphical files.

Input data required by Snappy, are a FASTA-file with the genome of the analyzed organism, and a bed-file, generated by “modkit pileup” (Fig. 1A). The modkit bed-file just summarizes the information from MM and ML fields in bam-files, so actually Snappy is compatible with any sequencing and data processing techniques that could provide MM + ML fields in BAM. Snappy does not have additional algorithm hyperparameters and automatically optimizes the threshold values based on the input data.

The main output of Snappy includes (i) summary table, where all identified motifs are listed, (ii) results table, where for each identified motifs all its genomic occurrences and corresponding methylation levels are listed. Additionally, Snappy generates two types of plots, representing methylation level of the motifs and their localization (Fig. 1B). In controversial cases, these plots might be used to ensure the correctness of the inferred motifs. For advanced users, who are interested in a more complex analysis, Snappy saves filtered and extended bed-files used during the enrichment process, and regexp-formatted records for each motif so that users could conveniently operate Snappy results using instruments such as Pandas or Polars.

### 2.2 Snappy enrichment algorithm

**Figure 2.**
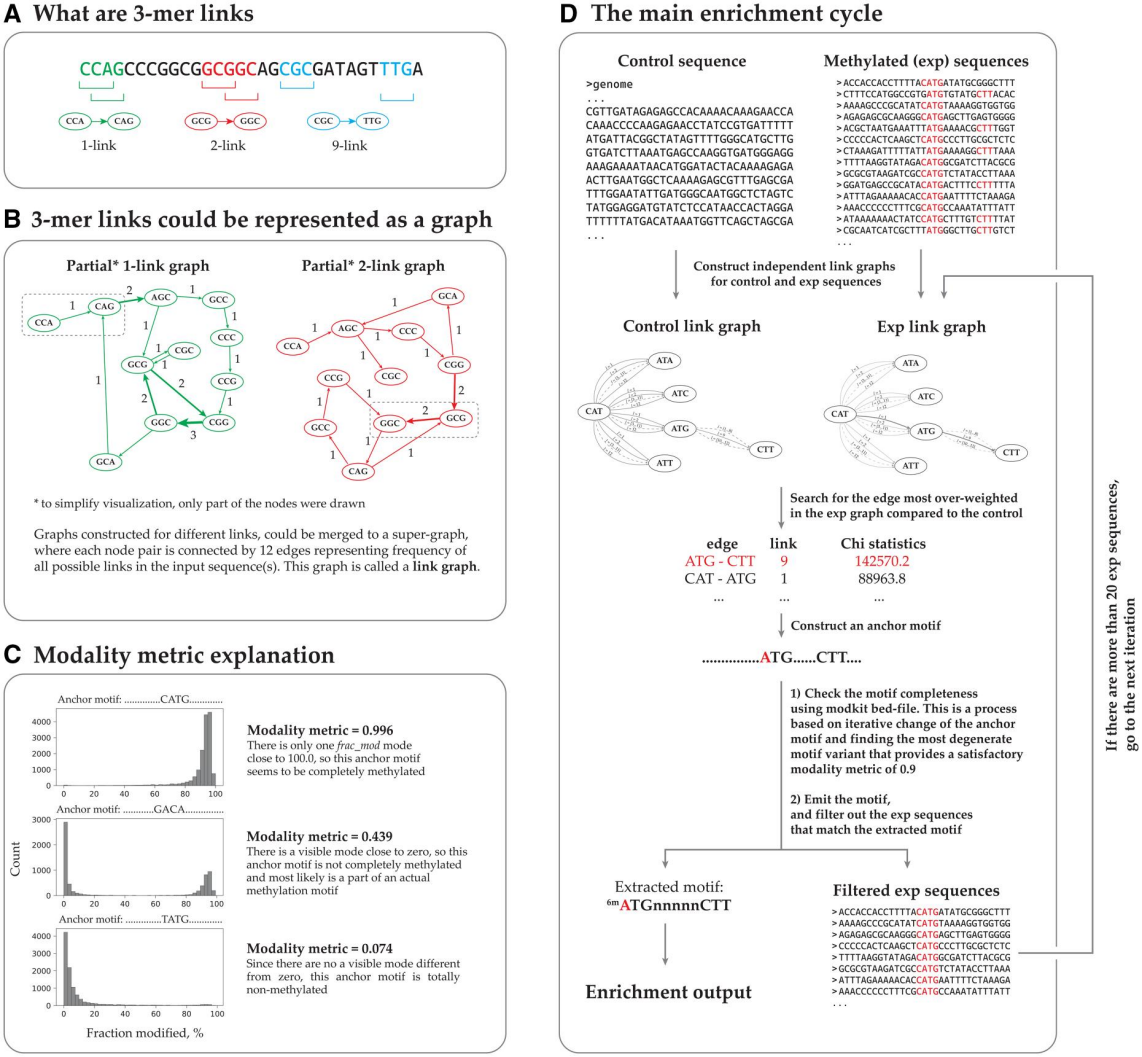
Enrichment algorithm explanation. (A) Definition of 3-mer links of different length. (B) Examples of 1-link and 2-link graph structures constructed based on the sequence from A. (C) Explanation of the modality metric used by Snappy to estimate the completeness of the motifs. In case when a motif is complete and totally methylated, the modality metric is expected to be close to 1.0. (D) A scheme of a single enrichment iteration.

The first step is enumerating all pairs of closely located 3-mers in the input sequence. In the algorithm description, such pairs are called links, and the distances between 3-mers in the link are called link lengths (Fig. 2A). Based on all links presented in the input sequence, Snappy constructs a graph structure, where each node represents a specific 3-mer, and each edge represents a link (Fig. 2B). Since the same 3-mers could be connected by links of different length, each node pair could be connected up to 12 labeled edges. Each edge in the graph is weighted, where edge weight equals the absolute abundance of this specific link in the input sequence. This complex graph is called a link graph.

Here we should define one more term called modality metric (Fig. 2C). In short, a modality metric is a value from 0.0 to 1.0 that indicates how completely a specific nucleotide motif is methylated. Snappy assumes that actual methylation motifs in bacteria are completely methylated, so it expects that modality metric for them is greater than 0.9. Modality metric is calculated based on “fraction modified” (or frac_mod) values assigned by ModKit for each genome position. These values are defined as the fraction of reads that bring a methylated base in a specific genome position. An intuitive explanation of the modality metric is available in Supplementary Data 1, available as supplementary data at *Bioinformatics Advances* online.

Snappy uses the genome of the analyzed bacteria as a negative background (control sequence), and genome contexts surrounding methylated positions as target sequences. Link graphs are constructed for both control and target sequences independently. Using Chi-square statistics, the algorithm searches for the link most over-weighted in the target sequences compared to the control, and uses it as an anchor motif. Next, the anchor motif is iteratively changed to find the most degenerate motif variant, which has a modality metric greater than 0.9. The resulting motif is added to the algorithm output, target sequences matching this motif are filtered out from the input data, and the algorithm goes to the next iteration trying to find another motif. The algorithm repeats the enrichment procedure until the number of target sequences becomes less than 20. A principal scheme of a single enrichment iteration is shown on Fig. 2D. A more formal algorithm description including details about anchor motif correction procedure is available in Supplementary Data 1, available as supplementary data at *Bioinformatics Advances* online.

## 3 Results

### 3.1 Method validation

The method was first validated on *H. pylori* J99 genome sequencing data, which was earlier characterized using both SMRT PacBio and Oxford Nanopore technologies (Krebes *et al.* 2014, Konanov *et al.* 2023). *H. pylori* is a unique bacterial species with a tremendous variety of restriction-modification (R-M) systems (up to 30 in some strains), so it has a very complex DNA methylation pattern, with a lot of overlapping or similar methylation motifs. Due to that, it was chosen as a validation object. Snappy was compared to the MicrobeMod pipeline, the only tool with similar functionality.

In result, Snappy outperformed MicrobeMod, demonstrating higher motifs enrichment accuracy for less computational time, and identified all known *H. pylori* J99 motifs. The only motif that was identified incompletely by Snappy was RTAYnnnnnRTAY, because its subvariants GTACnnnnnATAT and GTACnnnnnGTAC were not present in the reference genome, so the algorithm could not check if it was modified or not. All other RTAYnnnnnRTAY subvariants (RTAYnnnnnGTAT, RTATnnnnnRTAT, and ATAYnnnnnATAY) were successfully enriched, and the users can decide by themselves if these subvariants should be merged.

The results presented in Table 1 were obtained using all sequencing data with mean genome coverage depth of 565×. To estimate how sequencing depth affects enrichment accuracy, we performed a rarefaction test by random selection of the mapped reads (Supplementary Table S1, available as supplementary data at *Bioinformatics Advances* online). Based on the coverage depth tests, sensitivity and FDR (false discovery rate) curves were built (Supplementary Fig. 1, available as supplementary data at *Bioinformatics Advances* online). Most methylation motifs were correctly identified with coverage depth of 38×. The most difficult motifs to resolve were RTAYnnnnnRTAY, GGWCNA, and CCnnGG. It was noted above that RT^6m^AYnnnnnRTAY could be incompletely enriched due to its low prevalence in the genome. The GGWCN^6m^A motif had the same problem, because its GGTCGA subvariant has only four occurrences in the reference genome, which is not enough to provide satisfactory statistics, and correctness of the inference depended only on the order of anchor motifs selection.

**Table 1.** Identification of methylated motifs in *H. pylori* H99.

Modtype	reference motifs	MicrobeMod	Snappy	Snappy confidence
**^m6^A**	A**A**Gn_5_CTC	–	A**A**Gn_5_CTC	31 168.2
	A**A**Gn_6_TAAAG	–	A**A**Gn_6_TAAAG, CTTT**A**n_6_TTC	23 946.6, 25 388.7
	A**A**Gn_6_CTC	–	A**A**Gn_6_CTC, G**A**Gn_6_CTT	28 863.7, 17 374.2
	RT**A**Yn_5_RTAY	–	RT**A**Yn_5_GTAT, RT**A**Tn_5_RTAT, AT**A**Yn_5_ATAY^a^	105 38.0, 11 934.0, 5621.7
	C**A**TG	C**A**TG	C**A**TG	103 144.6
	G**A**TC	G**A**TC	G**A**TC	105 921.6
	G**A**GG	–	G**A**GG	66 960.1
	GCCT**A**	GCCT**A**nCRCY	GCCT**A**	55 102.7
	GWC**A**Y	GAC**A**CGM	GWC**A**Y	87 429.6
	G**A**NTC	–	G**A**NTC	109 180.8
	ATTA**A**T	–	ATTA**A**T	47 624.5
	GGWCN**A**	–	GGWCN**A**	84 397.9
	GT**A**C	–	GT**A**C	30 182.8
	GTS**A**C	–	GTS**A**C	5182.7
	TCG**A**	–	TCG**A**	6669.1
**^m4^C**	**C**CnnGG	**C**CMAGGCmmW	**C**CnnGG, **C**CBnGG	14 861.3, 58 207.1
	**C**CGG	**C**CGGnnnnnnnR	**C**CGG	86 867.7
	CGW**C**G	–	CGW**C**G	50 341.8
**^m5^C**	G**C**GC	G**C**GC	G**C**GC	133 960.6
	A**C**GT	MA**C**GTY	A**C**GT	105 417.1
	False positive	TGWC, AGCAATC, RMCCTC	–	
Execution time (without Modkit processing)		18 min 12 sec	7 min 25 sec	

a GTACn_5_ATAT/ATATn_5_GTAC/GTACn_5_GTAC were not present in the *H. pylori* J99 genome, that is why Snappy did not merge these motifs to RTAYn_5_RTAY.

Observed incompleteness of the ^4m^CCnnGG motif was the most intriguing. We found that when this motif intersects with motifs C^6m^ATG and ^4m^CCGG (in CCATGG and CCCGGG contexts respectively), it is totally unmethylated in the first cytosine (Supplementary Fig. 2, available as supplementary data at *Bioinformatics Advances* online). We suppose that it might be a consequence of competing between different MTases binding to the same genome sites. Again, completeness of the enrichment here depends on the starting anchor motif, that is why this motif could be extracted twice in a single run in both complete CCnnGG and incomplete CCBnGG/CCDnGG forms.

Next, we checked how the enrichment results depend on the chosen modkit threshold (—filter-threshold parameter). Five threshold values from 0.33 to 0.9 were tested. Snappy demonstrated very stable results (Supplementary Table S2, available as supplementary data at *Bioinformatics Advances* online) in all cases. We decided to use the value of 0.66 for full compatibility with “MicrobeMod call_methylation” output.

Finally, Snappy uses internal confidence threshold, which is actually the Chi statistics value indicating over-representation of the motif sequence in the modified contexts compared to the whole genome. The minimal confidence values that we observed for actual methylation motifs were 5182.7 and 6669.1 for GTSAC and TCGA motifs, respectively (Table 1, “Snappy confidence” column). However, to ensure sufficient sensitivity even for detecting very rare motifs, we decided to set the internal threshold at 100.0.

### 3.2 Identification of methylation motifs in other bacteria

To demonstrate versatility of the method, we additionally analyzed publicly available Oxford Nanopore sequencing data, for which POD5-files were available. Here, we first chose *Campylobacter* species for the analysis, since their genomes are also known to encode a lot of different R-M systems. Also, to demonstrate how the algorithm works with organisms with a less complex methylome, we ran Snappy on *Escherichia coli* and *Mycobacterium tuberculosis* genome sequencing data. *Campylobacter jejuni* ATCC 33560, *Campylobacter lari* ATCC 35221, *E. coli* ATCC 25922, and *M. tuberculosis* mc26030 raw sequencing data were obtained from The Melbourne University data repository (https://figshare.unimelb.edu.au/) and processed with both Snappy and MicrobeMod (Table 2). Where possible, the correctness of the identified motifs was checked using REBASE—a database for DNA restriction and modification enzymes (rebase.neb.com) (Roberts *et al.* 2023).

**Table 2.** DNA methylation motifs identified in external data with MicrobeMod and Snappy.

strain	MicrobeMod	Snappy
*Campylobacter jejuni* ATCC 33560 (coverage depth = 1915×)	AAATTT CATG RATATGnnWnW GACAATCA WGCGC	RA**A**TTY^a^ C**A**TG^a^ CAT**A**T^b^ GT**A**C^a^ GC**A**nnnnnnCTR^a^ ACA**A**YC^b^ RA**A**CnnnnnnRTTA^a^ CCYG**A**^a^ G**C**GC^a^
*Campylobacter lari* ATCC 35221 (coverage depth = 1319×)	AAATTT CYATnKnnnTTGY CMGGATCA CAAGTnnTWTAGMA AnAGCTGATCAA	RA**A**TTY^a^ CY**A**nnnnnnTTG^a^ G**A**TC^a^ GAR**A**nnnnnnnnTAC^c^ GTRG**A**G^b^
*Escherichia coli* ATCC 25922 (coverage depth = 406×)	GATC CCWGG	G**A**TC^a^ C**C**WGG^a^ RT**A**CnnnnGTG^a^ GAG**A**YC^d^ GGT**C**TC^a^^,d^
*Mycobacterium tuberculosis* mc26030 (coverage depth = 155×)	CTCCAGnnnSnnS	CTGG**A**G^a^

a Correctness of the sequences and presence of corresponding MTase genes in the genome were confirmed using REBASE.

b These motifs have been experimentally confirmed for the studied *Campylobacter* species using PacBio, but corresponding MTases are unknown (rebase.neb.com/cgi-bin/pacbioget?13650, rebase.neb.com/cgi-bin/pacbioget?42913).

c This motif is listed in REBASE as GADAnnnnnnnnTAC. We manually checked the methylation level of the GATAnnnnnnnnTAC subvariant, and did not observe a significant shift in frac_mod values (Supplementary Figure 3, available as supplementary data at *Bioinformatics Advances* online).

d “Y” degenerate base in GAGAYC is most likely an artifact, since its GAGATC subvariant contains a totally methylated GATC motif. Interestingly, its GAGACC subvariant methylated in adenine is a full complement for the GGTCTC motif methylated in cytosine. Non-symmetric methylation of GGTCTC/GAGACC has been shown earlier for *E. coli* strains (Bitinaite *et al.* 1992).

In *C. jejuni* 33560, all nine motif sequences identified by Snappy were complete and correct according to REBASE. Seven of them were confirmed using REBASE as well as presence of corresponding DNA MTases in the genome. Two other motifs also had records in REBASE for *C. jejuni* but were not linked to corresponding MTase genes (rebase.neb.com/cgi-bin/pacbioget?13650, rebase.neb.com/cgi-bin/pacbioget?42913). MicrobeMod returned only one correct motif sequence CATG, and four incompletely identified motifs.

In *C. lari* 35221, Snappy identified five methylation motifs. Four of them were confirmed using REBASE, but the GARAnnnnnnnnTAC motif identified by Snappy differed from the GADAnnnnnnnnTAC motif in REBASE by one degenerate base. We manually checked methylation calling results generated by Dorado for GATAnnnnnnnnTAC contexts and did not find a significant methylation level (Supplementary Fig. 3, available as supplementary data at *Bioinformatics Advances* online). For *C. lari*, none of the motifs returned by MicrobeMod were identified correctly.

In *E. coli* ATCC 25922, Snappy identified five methylation motifs. Four of them (GATC, CCWGG, RTACnnnnGTG, and GGTCTC) were in full accordance with REBASE records for this specific strain (tools.neb.com/genomes/view.php?seq_id = 90864&list = 1). The “Y” degenerate base in the motif GAGAYC was a result of superposition of actual *E. coli* methylation motifs GAGACC and G**A**TC, both methylated in adenine. Actually, such collisions could be resolved only experimentally. Additionally, in *E. coli* data we first faced systematic false-positive detection of methylation bases caused by neighborhood effects: the basecaller assigned quite high methylation probabilities for TC**C**nnCCTGG (in a form of TC**^5m^C**nnCCTGG), which was most likely caused by total cytosine methylation in the CCTGG submotif (Supplementary Fig. 4, available as supplementary data at *Bioinformatics Advances* online). MicrobeMod identified only two main methylation motifs—CCWGG and GATC.

In *M. tuberculosis* mc26030, Snappy identified only one methylation motif. According to REBASE, this strain has one more I type R-M system specific to the GCAYnnnnATC motif. We manually checked adenine methylation level in genome positions satisfying this motif and did not observe any adenine methylation signals (Supplementary Fig. 5, available as supplementary data at *Bioinformatics Advances* online). Probably, although the genes encoding this R-M system are present in the genome, it was not active in that specific sample. Also, the genome of *M. tuberculosis* contains a lot of poly-C regions, which has allowed us to detect a systematic homopolymeric effect, when Dorado assigns high methylation probabilities for cytosines located inside poly-C (Supplementary Fig. 6, available as supplementary data at *Bioinformatics Advances* online).

### 3.3 Performance tests

All procedures were conducted on the server with Ubuntu 22.04.5 LTS, equipped with two Intel Xeon Gold 6226R CPUs (64 threads), 1.48Tb RAM, and 3x NVIDIA RTX A5000 GPUs. Snappy analysis was performed in a dedicated conda environment with Python 3.12. In all cases, the most time-consuming process was Dorado basecalling, which totally utilized all available CPUs and GPUs.

Snappy utilized only a single CPU thread with maximum RAM usage linearly dependent on the genome size (Table 3). Snappy execution time was dependent on both genome size and a number of different methylation motifs. Generally, the Snappy algorithm is not demanding on computing resources and could be run on desktop PCs.

**Table 3.** Testing Snappy performance on bacteria with different genome sizes and number of methylation motifs.

Strain (genome size)	Number of methylation motifs	Execution time	Max. RAM usage
*Campylobacter lari* ATCC 35221 (1 513 369 bp)	3	3 min 58 sec	2144 Mb
*Campylobacter jejuni* ATCC 33560 (1 768 485 bp)	9	5 min 34 sec	2592 Mb
*Helicobacter pylori* J99 (1 655 304 bp)	20	7 min 25 sec	2402 Mb
*Mycobacterium tuberculosis* mc26030 (4 405 436 bp)	1	10 min 32 sec	4915 Mb
*Escherichia coli* ATCC 25922 (5 192 976 bp)	5	11 min 50 sec	6250 Mb

### 3.4 Main algorithm limitations

1. If a methylation motif has subvariants that are not presented in the considered genome, it could be extracted incompletely. Thus, *H. pylori* J99 actually has RTAY….RTAY methylation motif, but its subvariants GTAC….ATAT/GTAC….GTAC are not presented in the genome, so the algorithm cannot check if they are methylated or not, and returns as a result only RTAY….GTAT, RTAT….RTAT, and ATAY….ATAY subvariants. The users could decide by themselves if they should merge these subvariants.
2. If a motif is partially methylated in the genome, it could not provide satisfactory modality metric value, and could not be enriched by Snappy. In general, such cases are rather rare, and users are recommended to manually check motifs of the interest manually using modkit output, or by using additional enrichment methods like STREME if they suspect incomplete methylation.
3. If a motif has very few occurrences in the genome (typically less than 20) it cannot be identified by Snappy.

## 4 Methods

### 4.1. Acquisition of data used in the study

Genome sequencing of the *H. pylori* J99 genome was conducted during this study on the Oxford Nanopore platform (MinION) using an R10.4.1 flow cell (FLO-MIN114). *E. coli*, *M. tuberculosis*, and *Campylobacter* species sequencing data in the POD5 format were downloaded from the data repository of The University of Melbourne (https://figshare.unimelb.edu.au/).

### 4.2. Raw data preprocessing

First, POD5 files were processed by Dorado v0.7.1, with additional usage of models for 6 mA, 5mC, and 4mC (model versions v5.0). The resulting BAM files were converted to FASTQ using samtools (v1.19.2). FASTQ files were used for genome assembly with Flye (v2.8.1-b1676) (Kolmogorov *et al.* 2019). Using minimap2 (2.26-r1175), raw BAM files were remapped to the assembled genome to obtain sorted mapped BAM files with MM and ML fields. These files were processed by Modkit (v0.2.4) with a parameter—filter-threshold 0.66 to obtain bed-files next passed to Snappy.

## 5 Discussion and conclusion

The most recent r10.4.1 version of Oxford Nanopore Technology flow cell and new basecalling models significantly increase accuracy of methylated bases detection and simplify the analysis. Despite that, algorithms currently used for identification of methylation motifs continue to rely only on nucleotide sequences surrounding modified bases but do not use base modification information in SAM auxiliary tags (MM/ML), which could significantly enhance motif identification accuracy. In our knowledge, Snappy presented in this study is the first attempt to combine enrichment algorithms with simultaneous analysis of modification probabilities provided by basecaller Dorado.

Snappy successfully identified all methylation motifs in genomes of *H. pylori* and two *Campylobacter* species, thus demonstrating high accuracy in motif extraction on the organisms with a complex methylome. DNA methylation motifs in bacteria species with fewer R-M systems such as *E. coli* and *M. tuberculosis* were successfully identified as well.

Compared to Snapper designed to work with r9.4.1 flow cell data, Snappy does not use any enrichment heuristics, and does not require control sample sequencing. That, coupled with a new graph-based enrichment algorithm, provides very high processing speed. Moreover, due to a new logic, Snappy enriches long bipartite motifs as easily as short motifs and uses the same confidence metrics for all motifs, which simplifies the results interpretation. Finally, although Snappy was primarily designed to process ONT data, it is also capable of working with any BAM-files with MM/ML tags.

Snappy allowed us to reveal two types of artifacts arising on the basecalling stage, which could affect DNA methylation analysis. First, we faced the neighborhood effect when positions very close to modified bases were also systematically identified by Dorado as modified. Second, although new Oxford Nanopore Technology chemistry significantly improves resolution of homopolymers, we found that short homopolymers could lead to false positive inference of modified positions. Thus, Snappy has highlighted the ways in which current non-canonical Dorado models can be improved.

## Supplementary Material

vbaf296_Supplementary_Data

## Data Availability

The *H. pylori* J99 reads in BAM-format are available in BioProject PRJNA1301222. POD5-files for other bacteria used in the study are available in the repository of The Melbourne University: figshare.unimelb.edu.au/articles/dataset/ATCC_33560_202309_pod5_data/25495054 figshare.unimelb.edu.au/articles/dataset/ATCC_35221_202309_pod5_data/25493905 figshare.unimelb.edu.au/articles/dataset/ATCC_25922_202309_pod5_data/25521892 figshare.unimelb.edu.au/articles/dataset/AMtb_1_202402_pod5_data/25495045
